# Changes in Student’s Breakfast and Snack Consumption during the Second COVID-19 Lockdown in Portugal: A Five-Wave Study

**DOI:** 10.3390/ijerph20043034

**Published:** 2023-02-09

**Authors:** Paula Magalhães, Beatriz Pereira, Francisco Garcia, Catarina Vilas, Tânia Moreira, Pedro Rosário

**Affiliations:** School of Psychology, University of Minho, 4710-052 Braga, Portugal

**Keywords:** breakfast, snacking, lockdown, COVID-19, children, students, feeding patterns, healthy eating index, healthy behavior promotion

## Abstract

The circumstances of the COVID-19 lockdown affected many students’ life spheres, including their feeding patterns and snack intake. The main goals of the present study were to: (a) analyze the changes in students’ breakfast and snacking consumption during lockdown, and (b) analyze changes in the content of the students’ snacks using the Healthy Eating Index. This study analyzed data from a sample of 726 students from 36 classes from the late elementary (i.e., fifth grade) through high school (i.e., twelfth grade) from two public schools in the north of Portugal. Data were collected in five moments during the 2020/2021 school year, pre-, during, and post-second lockdown moments. Throughout the five moments, almost 90% of the students ate breakfast, and the majority brought snacks from home to eat in school. Surprisingly, there was an increase in the quality of the snacks consumed during lockdown compared to the pre-lockdown moments (e.g., consumption of more whole and total fruits and less consumption of food with added sugar, saturated fats, refined grains, and fatty acids). Suggestions for healthy behavior promotion will be discussed, such as improving the school food environment and teaching children to prepare healthy lunch boxes.

## 1. Introduction

Healthy behavior promotion, such as the promotion of a healthy diet, is a top public health priority to prevent chronic diseases (e.g., obesity, diabetes, heart disease, and cancer), and to guarantee a healthy lifestyle from early childhood into late adulthood [1]. The literature highlights that eating habits promoted during childhood continue throughout adulthood [2], which stresses the importance of promoting healthy eating behaviors early in life. 

Extant studies pointed out two major themes to be considered in the healthy eating behaviors domain: breakfast [3] and snacking [4,5]. Skipping breakfast seems to be the most critical risk factor for overweight and obesity among adolescents [3]. Contrarily, taking breakfast is crucial for school performance, and is associated with better concentration [6], memory, attention, and less frustration [7]. Moreover, breakfast consumption is positively associated with socioeconomic status (SES) [8], and seems to be more common in boys [9]. In European countries, it appears that most children take breakfast regularly (e.g., in Italy, only 10.8% of the children skip breakfast) [10,11], while in Saudi Arabia, it is more common for children to skip this meal [8].

Similar to breakfast consumption, there is a positive association between snack intake and well-being in children and adolescents [4,5]. Overall, snacking can be defined as consuming small quantities of food between main meals [4,5,12,13]. The popularity of snacking has been increasing over time, making snacks an essential source of energy intake [5,13]. A cross-cultural study showed that snacking patterns vary from country to country [4]. For example, the percentage of snacks consumed was higher in Australia and US, followed by Mexico, and lower in China. In addition, snacking varies across different age groups, with children aged two to five years old having the highest rate of snacking (i.e., 79%) compared to other age groups [14]. The type of snacks also varies across age groups. For example, for children aged four to eight years old, the snacks consumed are typically dairy and fruit, whereas the preferences of children from nine to 13 years old go from savory snacks to sweetened beverages [15]. In fact, there seems to be a transition between these age groups’ food preferences. A study from Italy showed that only 23.7% of children, approximately eight years old, ate at least one fruit or had a fruit juice as a snack [10]. Another study also showed that amongst children over ten years old, the most preferred snacks were sweetened coffee and tea, sweets and desserts, fruit, sugar-sweetened beverages, and high-calorie finger foods (fried/baked dough with meat/cheese/vegetables) [16]. Notably, adolescents (12–18 years) were the only ones who did not make fruits the most common snack [14]. Moreover, in a recent study, the students (aged 11–13 years) who reported snacking more frequently preferred high-sugar and high-calorie snacks over whole-grain snacks [17]. Finally, among adolescents, snack frequency was inversely associated with diet quality, i.e., at this age, additional snacks decreased the diet quality [18]. To summarize, adolescents seem to make fewer healthy choices than younger children do [5,15].

Other relevant factors should be considered when analyzing snacking behavior, such as where the snack was acquired, the students’ sex, and their socioeconomic status (SES). For example, Johnston et al. [19] found that homemade meals were less healthy than those that were prepared in schools. Overall, homemade meals are highly processed [20] and contain less fruit, vegetables, and dairy than meals served in school [19]. However, adolescents are also more likely to choose unhealthy snacks when those types of food are readily available in the school cafeteria or vending machines [21]. Regarding the variable sex, research shows that boys consume more snacks than girls [21]. Finally, students from higher SES take more servings of snacks per day than those from lower SES [22].

The year 2020 was marked by the spread of the coronavirus disease. Lockdowns were mandatory, with changes in daily life routines, from educational to social activities [23,24]. Schools closing was also mandatory, affecting students’ life; for instance, there was a shift to online or at-distance classes, with the need for additional resources, such as computers, television, or radio broadcast [25,26]. Staying at home also changed students’ eating habits. A study conducted in Greece suggested that children’s breakfast consumption during the first lockdown period increased in 5% [27]. In addition, there was a higher percentage of snack consumption (e.g., vegetables, dairy, sugary foods) overall, and a rise in food cravings during the lockdown compared to the period previous to the lockdown [28]. For example, a study by Sidor and Rzymski [29] found that 52% of individuals snacked more during the lockdown. Additionally, the lockdown period was associated with low consumption of vegetables, fruits, and legumes, and high adherence to potato chips, meat, dairy, sugary drinks, and fast food [29]. In fact, during the pandemic, North Americans felt more motivated to consume sweets and fast food, being capable of paying more, waiting more, and working harder for these foods than for vegetable and savory snacks [30]. Rodríguez-Pérez et al. [31] found a distinct trend in Spain; data showed that, during the lockdown, adherence to a Mediterranean Diet (i.e., olive oil, vegetables, fruit, low protein) [32] increased, and the intake of pastries and sweetened beverages decreased. Similar results were found in Greece, another European country following a Mediterranean Diet, where children decreased fast-food consumption and increased fruit and vegetables intake due to parents home-cooking more often during the lockdown [27].

Similar to some of the aforementioned results, a study by von Hippel and Workman [33] showed that, during summer vacations, students increased their energy intake. This suggests that school may be a protective factor against unhealthy eating behavior [33]. As it occurs in summer, during the lockdown students may have faced similar difficulties in maintaining healthy behaviors due to a high level of sedentary behavior and an increase in hungriness due to stress [34]. Another contributing aspect to this trend could be that when students find snack foods available at home, they are more likely to consume them more frequently [35,36]. This is important because healthy eating affects individuals’ general well-being [37].

### Study’s Purpose

Considering the changes in the school routines imposed by the COVID-19 pandemic (e.g., school cafeterias were partially or fully closed even after schools started working post-lockdown, interfering with children’s snack consumption) [38], the main purpose of the present study was to learn to what extent children’s breakfast and snacking patterns have changed throughout pre-, during, and post-second lockdown in Portugal. This purpose was divided into two major goals that guided the study: (i) The first goal aimed to evaluate the frequency of breakfast consumption and snacking, and to map where the snack was acquired from (i.e., home, bought in school, or both) across the school year (i.e., examining possible changes during pre-, during, and post-second lockdown); and (ii) the second goal aimed to understand how the content of the snacks changed across time, especially differences between pre-, during and post-second lockdown moments. 

The present study’s novelty arises from the longitudinal design that compares breakfast and snacking data from pre-, during, and post-second COVID-19 lockdown periods. Only considering data from these three moments it is possible to evaluate the real impact of the lockdown on students’ eating patterns. Understanding the effects of the pandemic-related lockdown on Portuguese students’ breakfast and snacking habits throughout a school year will contribute to adjusting breakfast and snacking guidelines, and help parents and children make appropriate food choices in future similar or parallel situations (e.g., summer vacations) [4].

To address the present student’s goals, the current investigation involves a five-wave longitudinal study with students from the fifth to the twelfth grades across a school year (2020–2021), covering in-person school attendance and lockdown periods. 

## 2. Materials and Methods

### 2.1. Participants

**Figure 1:**
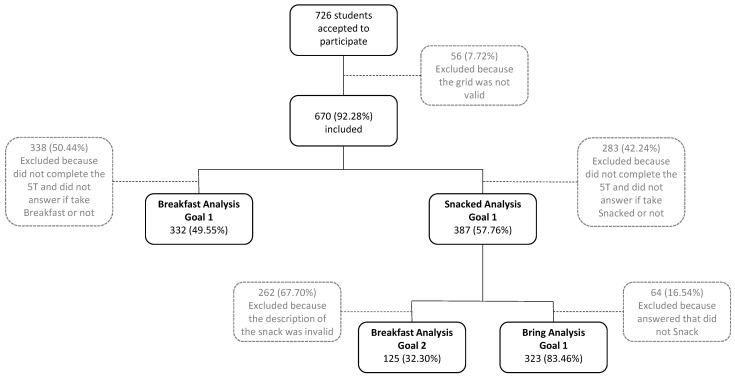
Flowchart of the participants’ inclusion/exclusion process for each study goal: breakfast consumption, snacking, and bring analysis (i.e., where the snack was acquired) for the first goal; qualitative analysis for the second goal (black boxes refer to the participants included in the study, and the grey ones refer to participants excluded from the study).

Data were collected from a sample of 726 students from 36 classes of the late elementary (i.e., fifth grade) through high (i.e., twelfth grade) school, from two public schools in the north of Portugal. Ages ranged from 9 to 19 years (*M* = 12.82; *SD* = 2.03), and 382 were males (52.6%). Of the initial pool of participants, for each goal, only participants that provided the required information for the specific goal across the five moments were included in the subsequent analysis. The rationale for this choice was to guarantee the comparison of the same students across moments. The inclusion process relied on the goals of the present study (see Figure 1 for a depiction of the inclusion process). 

To answer the first goal (i.e., frequency of taking breakfast and snacking, where the snack was acquired-home, school, or both –, and how it changed across the five moments; see Figure 1), three subsamples of participants were devised based on whether the participants answered if they (i) had breakfast, (ii) had snacked, and (iii) had brought the snack from home, bought it at school or both. Regarding this goal, the subsample (i) included 332 students, 169 boys (50.9%), ages ranging from 9 to 18 years (*M* = 12.81; *SD* = 1.99). The subsample (ii) included 387 students, 210 boys (54.3%), ages ranging from 9 to 18 years (*M* = 13.13; *SD* = 1.80); and the subsample (iii) included 323 students, 163 boys (50.5%), ages ranging from 9 to 18 years *(M* = 13.04; *SD* = 1.81). 

To answer the second goal (i.e., understand the content of the snacks and how it changed across the five moments), participants reporting having snacked and whose responses correctly reported the content of their snacks were included. The subsample for this goal included 125 students, 54 Boys (43.2%), ages ranging from 9 to 18 years (*M* = 12.68; *SD* = 1.94).

### 2.2. Instruments and Measures

#### 2.2.1. Sociodemographic Questionnaire

To characterize the participants, information about the age, sex, grade, and family income of each student was collected. Family Income typically refers to the value or amount of money an individual or a group receives for their work [39], and it bears a vital role in food purchases [40]. We accessed the School Social Action Level (SSAL) to evaluate the family’s income. In Portugal, SSAL corresponds to the sum of the income of all household members per year. It is divided into three levels: ‘A’ corresponds to a family’s income of up to 3071.67EUR, ‘B’ corresponds to a family’s income of up to 6143.34EUR, and ‘C’ corresponds to a family’s income of up to 9215.01EUR. An income superior to the level ‘C’ value corresponds to a family that does not have SSAL and is considered a high income [41]. In our main sample, 14.2% of the students were allocated to level ‘A’ (N = 103), 23.1% to level ‘B’ (N = 168), 16.5% to level ‘C’ (N = 120), and 46.1% (N = 335) did not benefit from any governmental help.

#### 2.2.2. Breakfast and Snacking Observational Grid

**Figure 2:**
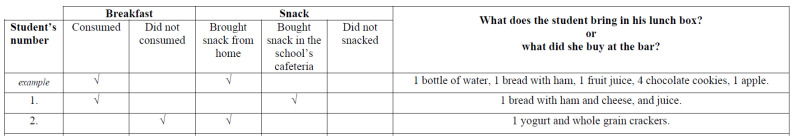
Example of a part of an observational grid.

An observational grid was used to evaluate the student’s breakfast and snacking patterns and quality during the school year (see Figure 2). The following topics were asked on the observational grid: (i) if students had breakfast; (ii) if students snacked; (iii) if the snack was brought from home, bought in the school’s cafeteria, or both; and (iv) identification of the content of the student’s snack. Note that the topic regarding breakfast consumption was only included from T2 onwards after an update of the literature review.

The first three indicators were codified on a dichotomous scale (yes/no), and the frequency of each behavior was considered. The fourth indicator was codified based on the head teachers’ written responses about the content of students’ snacks, specifically the type of foods and the quantities consumed (see Data Analysis-second goal).

### 2.3. Procedure

The present study is part of a research project that has been approved by the University of Minho Ethics Committee for Research in Social and Human Sciences (CEICSH) (CEICSH 032/2019). CEICSH considered that the project complies with the requirements for good practice in human research, in accordance with national and international standards governing research in social and human sciences, including the Declaration of Helsinki. 

**Figure 3:**
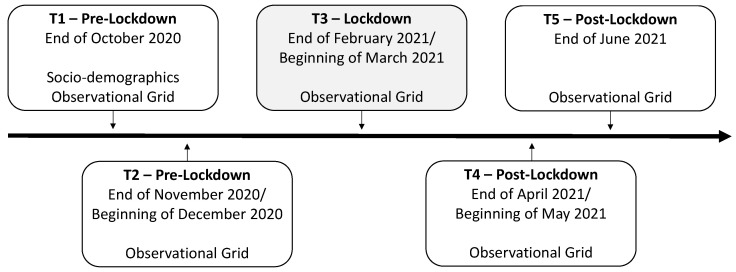
Data collection timeline: time frame for each data collection (T1–T5), classification into pre-, during, and post-second lockdown, and instruments collected in each moment.

According to the goal of understanding the changes in students’ breakfast and snack consumption pre- during, and post-second lockdown, the present study followed a longitudinal design with five moments. Figure 3 summarizes the five moments (T1–T5) of data collection. Note that the goal of the study was to analyze patterns of change across a school year characterized by several restrictions due to the pandemic (e.g., social distancing). To this purpose, we decided to conduct a five-wave longitudinal study. Coincidently, a lockdown occurred around the third moment (T3) of data collection, which allowed us to understand the impact of this public health measure on students’ behavior. 

Prior to data collection, students and parents/caregivers were informed by the research assistants about the aims of the study and were assured of the confidentiality of the data. Moreover, to protect the confidentiality and anonymity of the data, codes were assigned to identify the participants. Children’s and parents/caregivers’ written informed consent agreements were requested. Then, data collection took place in regular classes. The class head teacher collected the data by asking students individually: (i) did you have breakfast today?; (ii) are you having a snack today? Did you bring it from home or buy it in the school cafeteria?; and (iii) can you show me your lunchbox or what you bought in the school cafeteria? Based on students’ responses and on the content of their snacks, the class head teachers filled in the observational grid (see Section 2.2). For each class, per moment (T1–T5), data was collected on a single day.

### 2.4. Data Analysis

#### 2.4.1. First Goal: Frequency Analysis of Taking Breakfast, Snacking, and Where the Snack Was Acquired—Home, School, or Both—And Learn How It Changed across Pre-, during, and Post-Second Lockdown Moments

A non-parametrical statistical test called Cochran’s Q test was used to analyze general frequency differences among the five moments (T1–T5). This analysis was chosen due to the dichotomous nature of the responses. Then, McNemar test was used to compare pairing means. The Generalized Estimating Equations (GEE) was also computed to search for main patterns across the moments. The analysis regarding where the snack was acquired from was represented by three possible responses (home, school, and both), and consisted of the frequency and descriptive analysis. The data analysis was computed using the IBM^®^ SPSS^®^ Statistics, version 27.0 for Microsoft^®^ Windows^®^ (Armonk, NY, USA).

#### 2.4.2. Second Goal: Content of the Snacks Analysis and How It Changed across Moments

The codification of the content of the snacks was based on the Healthy Eating Index (HEI) categories [42]. The HEI is a comprehensive index comprising 13 food components, both healthy (e.g., total fruits) and unhealthy (e.g., added sugars), reflecting the key recommendations of the 2015–2020 Dietary Guidelines for Americans. Additionally, this is a validated and recommended index for research purposes. The food components are: total fruits, whole fruits, total vegetables, greens and beans, whole grains, dairy, total protein foods, seafood and plant proteins, fatty acids, refined grains, sodium, added sugars, and saturated fats. In the present study, the frequency of each component per each student’s snack was codified (see Table 1 to see components and respective examples).

To analyze the frequency of each HEI component, the number of portions of each component contained in a snack was quantified. To calculate the number of portions, we used the Food Patterns Equivalents Database (FPED) [43]. FPED converts the foods and beverages into portions (e.g., cups, teaspoons) of components that are similar (e.g., fatty acids and saturated fats vs. solid fats) to the HEI ones. Lastly, the FPED database does not include the category sodium, so the database Food Composition Table [Tabela de Composição de Alimentos] [44] was used to codify this component. This database indicates the snacks that contain sodium in grams per 100 g of edible parts. Therefore, snacks including more than 0.3 g of salt were considered as one portion of sodium, as indicated in the Guide to Healthy School Snacks [Guia Para Lanches Escolares Saudáveis] [45]. 

Before the coding of the student’s snacks, two researchers trained the coding scheme as described next: (i) The researchers first discussed the distinguishing features of each HEI component; (ii) researchers practiced together applying the codebook to a series of snacks until they agreed on coding; (iii) each researcher independently coded ten snacks during practice, compared scores, and resolved any differences through discussion; and (iv) training continued until both researchers assigned HEI components that differed by no more than one snack in ten consecutive trials. Once the criteria were met, one researcher scored all the snacks of the sample, and the other researcher independently scored 30% of those snacks. Interrater reliability between the two researchers was 0.87.

After coding the content of the snacks, the number of portions of each HEI component was compared across time. Specifically, Repeated Measures ANOVA was used to compare the frequency of the components across time (i.e., T1–T5). After determining differences between moments, post hoc comparisons were conducted to understand which moments the differences occur. The data analysis was computed using the IBM^®^ SPSS^®^ Statistics, version 27.0 for Microsoft^®^ Windows^®^.

## 3. Results

### 3.1. First Goal-Frequency Analysis of Taking Breakfast, Snacking, and Where the Snack Was Acquired-Home, School, or Both-, and How It Changed across Pre-, during, and Post-Second Lockdown Moments

#### 3.1.1. Descriptive Statistics

Table 2 provides an overview of the data, presented in absolute (n) and percentages (%). It shows three major variables, i.e., breakfast, snack, and where the snack was acquired from, across the five moments (i.e., T1–T5). Furthermore, data are organized by the variable sex (i.e., male and female) and school year; school years were grouped according to the Portuguese school cycles (i.e., 5th–6th, 7th–9th, and 10th–12th).

#### 3.1.2. Breakfast Analysis

Considering the total sample, around 90% of the students took breakfast at every moment: 90.7% in T2, 91.4% in T3, 89.7% in T4, and 90.4% in T5 (see Table 2). Cochran’s Q test indicated no statistically significant differences among the four moments. Moreover, there was no significant statistical difference regarding sex, school cycle, and income level, χ2(3) = 2.59, *p* = 0.46. 

#### 3.1.3. Snacking Analysis

Data showed that most students snacked: 92.8% in T1, 90.6% in T2, 80.6% in T3, 88.3% in T4, and 85.8% in T5 (Table 2). Cochran’s Q test indicated differences among the five moments, χ2(4) = 18.79, *p* < 0.001. Post hoc McNemar’s test with manual Bonferroni correction showed that: (i) the consumption of snacks at T1 (i.e., pre-second lockdown; frequency = 92.8%) was higher than that of T3 (i.e., during second lockdown; frequency = 80.6%) and higher than that of T5 (i.e., post-second lockdown; frequency = 85.9%); (ii) the consumption of snacks at T2 (i.e., pre-second lockdown; frequency = 90.6%) was higher than that of T3 (i.e., during second lockdown; frequency = 80.6%); and (iii) the consumption of snacks at T3 (i.e., during second lockdown; frequency = 80.6%) was smaller than that of T4 (i.e., post-second lockdown; frequency = 88.3%) (see Table 3). 

Results showed differences in snack consumption regarding sex, χ2(1) = 13.47, *p* < 0.001, with girls reporting snacking more than boys (OR > 1). Moreover, there were also differences in snack consumption regarding the school grades χ2(2) = 27.19, (*p* < 0.001). Pairwise comparisons indicated that students from 5th–6th grades reported having snacked more than students from the 7th–9th grades (*p* = 0.000), and more than students from the 10th–12th grades (*p* = 0.036) (OR < 1) (see Table 4). Finally, there were no significant differences in snack consumption regarding income, χ2(3) = 4.07, (*p* = 0.25).

#### 3.1.4. Where the Snack Was Acquired Analysis

Results indicated that at T1 most participants (90%) brought the snacks from home, at T2, 91.3%, at T4, 90.6%, and at T5, 85.1%. Note that there are no data regarding T3 due to students being at home. Between 4.5% and 10.4% of the students exclusively bought snacks at school, and a residual number of students brought snacks from home and bought snacks at school (i.e., between 1.3% and 1.9%) (see Table 2).

### 3.2. Second Goal-the Content of the Snacks Analysis and How It Changed across Moments

#### Content of the Snacks

**Figure 4:**
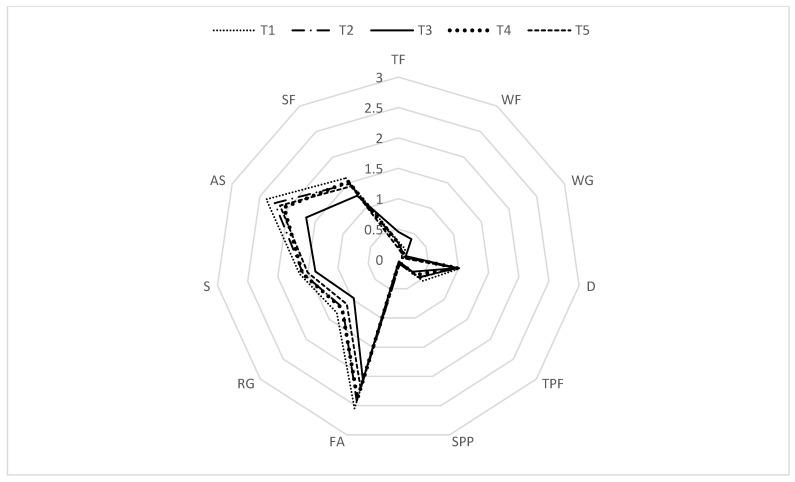
Graphic for the HEI components consumed among moments. Each angle of the graphic represents a distinct component (i.e., TF—Total Fruits, WF—Whole Fruits, WG—Whole Grains, D—Dairy, TPF—Total Protein Foods, SPP—Seafood and Plant Protein, FA—Fatty Acids, RG—Refined Grains, S—Sodium, AS—Added Sugars, SF—Saturated Fats), and a distinct line represents each moment. Unity of measures = portions.

Figure 4 shows all HEI components consumed in every moment (i.e., T1–T5).

Overall, across the five moments, the most prevalent component present in the snack choices was fatty acids (e.g., white bread), followed by added sugars (e.g., cookies), sodium (e.g., ham), and saturated fats (e.g., butter). The components total vegetables and greens and beans did not take part in these students’ snacks. Data on each component and differences across moments will be further described (see Table 5 and Table 6).

Regarding the component total fruits, the means of portions consumed across moments differ significantly: F (3.77, 467.93) = 7.26, *p* < 0.001. Mauchly’s test indicated a violation of sphericity [χ2(9) = 24.15, *p* < 0.001]; thus, post hoc comparisons using a *t*-test with Bonferroni correction were conducted. Results indicated that the consumption of total fruits was significantly higher during the second lockdown period (i.e., T3, *M* = 0.45, *SD* = 0.57) compared to one of the pre-second lockdown moments (i.e., T2 *M* = 0.27, *SD* = 0.48), and to both post-second lockdown moments (i.e., T4, *M* = 0.27, *SD* = 0.48; T5, *M* = 0.18, *SD* = 0.40). Thus, the consumption of total fruits increased during the second lockdown period and decreased when students returned to school. 

Similarly, the means of portions of whole fruits consumed across moments differ significantly: F (2.81, 348.98) = 16.37, *p* < 0.001. Mauchly’s test indicated a violation of sphericity [χ2(9) = 99.37, *p* < 0.001]; thus, post hoc comparisons using a *t*-test with Bonferroni correction indicated that the mean of portions of whole fruits consumed during T3 (*M* = 0.40, *SD* = 0.55) was significantly higher than that at T1 (*M* = 0.20, *SD* = 0.44), T2 (*M* = 0.14, *SD* = 0.34), T4 (*M* = 0.14, *SD* = 0.34), and T5 (*M* = 0.11, *SD* = 0.32). Again, the consumption of whole fruits also increased during the second lockdown period and decreased when students returned to school.

Data showed that, for both whole grains [F (3.64, 451.61) = 1.02, *p* = 0.394] and dairy [F (3.69, 457.32) = 0.17, *p* = 0.945], there were no statistically significant differences in the number of portions consumed across moments. Note that Mauchly’s test also indicated a violation of sphericity for these two components [χ2(9) = 36.60, *p* < 0.001 and χ2(9) = 31.37, *p* < 0.001, respectively].

Regarding total protein foods, the differences between the mean of portions consumed across moments were statistically significant: F (3.71, 460.26) = 3.45, *p* = 0.01. Mauchly’s test indicated a violation of sphericity [χ2(9) = 25.20, *p* = 0.003]: thus, post hoc comparisons using the t-test with Bonferroni correction indicated that the mean of portions of total protein foods consumed during the pre-second lockdown moment (i.e., T1, *M* = 0.54, *SD* = 0.65) was significantly higher compared to the mean of portions consumed during the second lockdown moment (i.e., T3, *M* = 0.30, *SD* = 0.50). Finally, the consumption of total protein foods decreased during the second lockdown period, with the values being maintained after students returned to school.

Data indicated no statistically significant differences in the number of portions of seafood and plant protein consumed across moments: F (3.46, 429.59) = 1.27, *p* = 0.282. Note that Mauchly’s test indicated a violation of sphericity [χ2(9) = 43.73, *p* < 0.001].

Regarding fatty acids, the differences between the mean of portions consumed across moments were statistically significant: F (3.77, 466.97) = 5.62, *p* < 0.001. Mauchly’s test indicated a violation of sphericity [χ2(9) = 30.98, *p* < 0.001]; thus, post hoc comparisons using the t-Test with Bonferroni correction indicated that the mean of portions of fatty acids consumed during T3 (*M* = 2.06, *SD* = 1.10) was significantly lower compared to the mean of portions consumed during T1 (*M* = 2.55, *SD* = 1.08) and T2 (*M* = 2.42, *SD* = 1.07). Thus, the consumption of fatty acids decreased during the second lockdown, with the values being maintained during the post-second lockdown period. 

Moreover, the consumption of refined grains was also significantly different across moments: F (3.78, 469.26) = 10.24, *p* < 0.001. Mauchly’s test indicated a violation of sphericity [χ2(9) = 26.42, *p* = 0.002]; post hoc comparisons using the t-test with Bonferroni correction indicated that the mean of portions of refined grains consumed during T3 (*M* = 0.97, *SD* = 0.54) was significantly lower than the mean of portions consumed during T1 (*M* = 1.34, *SD* = 0.62), T2 (*M* = 1.21, *SD* = 0.51), and T4 (*M* = 1.22, *SD* = 0.57). In addition, there was also a significant difference between the mean of portions of refined grains consumed during T1 (*M* = 1.34, *SD* = 0.62) compared to the portions consumed during T5 (*M* = 1.12, *SD* = 0.53). For refined grains, the consumption decreased during the second lockdown period and increased when students returned to school; however, the consumption does not seem stable across time (i.e., during T5, the consumption of refined grains seems to be lower compared to the pre-second lockdown moment). 

Regarding sodium, there were no statistically significant differences in the number of portions consumed across moments: F (3.8, 471.23) = 1.55, *p* = 0.19. Note that Mauchly’s test indicated a violation of sphericity [χ2(9) = 22.67, *p* = 0.007]. 

Regarding added sugars, the differences between the mean portions consumed across moments were statistically significant: F (4, 496) = 11.97, *p* < 0.001. Mauchly’s test indicated no violation of sphericity [χ2(9) = 13.83, *p* = 0.13]; Post hoc comparisons indicated that the mean of portions of added sugars consumed during T3 (*M* = 1.66, *SD* = 0.99) was significantly lower compared to the mean of portions consumed during T1 (*M* = 2.38, *SD* = 1.05), T2 (*M* = 2.23, *SD* = 0.98), T4 (*M* = 2.06, *SD* = 0.98), and T5 (*M* = 2.14, *SD* = 0.99). Thus, students consumed less added sugars during the second lockdown period compared to pre-second and post-second lockdown moments. 

Lastly, the mean of portions of saturated fats consumed across moments differs significantly: F (3.89, 481.59) = 4.153, *p* = 0.03. Mauchly’s test indicated a violation of sphericity [χ2(9) = 17.66, *p* = 0.04]; post hoc comparisons using the *t*-test with Bonferroni correction indicated that the mean of portions of saturated fats consumed during T3 (*M* = 1.25, *SD* = 0.76) was significantly lower compared to the mean of portions consumed during T1 (*M* = 1.59, *SD* = 0.80) and T4 (*M* = 1.52, *SD* = 0.74). The consumption of saturated fats seems to have decreased during the second lockdown period and increased after students returned to school.

## 4. Discussion

The present study goals were twofold. We aimed to understand the impact of the second COVID-19 lockdown on students from the fifth through the twelfth grades’ breakfast and snacking patterns (i.e., first goal) and on the content of the snacks consumed (i.e., second goal). These goals were addressed using an observational grid across five moments throughout a school year. This study comprised a period of lockdown (i.e., T3) and periods of regular school time (i.e., T1, T2, T4, and T5). For the first goal, we analyzed whether students ate breakfast and snacked, and mapped where the snack was acquired. For the second goal, the observational grid included information on the content of the students’ snacks across the school year. To this aim, the snacks were codified based on a global, standardized, and widely used measure-the HEI.

Regarding the first goal, results showed that around 90% of students took breakfast across the five moments. The habit of taking or skipping breakfast seems to be related to cultural norms, with European countries valuing regular breakfast consumption (e.g., Nelson et al. [11]). For example, a study conducted in England showed similar results to the present ones, indicating that less than 10% of students aged four to 18 skipped breakfast. Distinctively, a study conducted in Saudi Arabia showed that 79% of children skipped breakfast [8].

In the present study, breakfast consumption patterns seem to be similar regarding sex, school grades, and income. Regarding the variable sex, the WHO reported that girls are likely to skip breakfast more frequently than boys [9]. However, a recent study showed similar results to the current data (i.e., no differences between boys and girls) [8], suggesting that the sex disparities in breakfast consumption may have been attenuated over the years. Regarding students’ school grades, the literature indicates that breakfast consumption tends to decrease with age [9]. The present study focused on late childhood and adolescence; therefore, current data may not be sensitive enough to detect changes in breakfast patterns when comparing younger children with adolescents and young adults. Regarding income, the literature suggests that children with higher SES consume breakfast more frequently than children with lower SES [8,9,46]. The present study did not find such differences; the current result may mirror Portuguese cultural norms regarding the importance of breakfast consumption. For example, these policies have been translated into breakfast incentive policies [47,48], such as school efforts to deliver breakfast to children with low SES. 

Similar to the breakfast data, most of the sample snacked in all five moments; still, the proportion of students reporting to have snacked decreased over time. Interestingly, more students reported having snacked before the second lockdown than during the lockdown. Current data are inconsistent with the prior literature suggesting that lockdown affected people’s eating patterns [30]; for example, the work by Sidor and Rzymski [29] associated the lockdown experience with increased snack consumption. However, despite this contradictory evidence, Rodríguez-Pérez et al. [31] found a decrease in snack consumption during the COVID-19 lockdown. This evidence is interesting because the population of this study is culturally similar to the Portuguese one (i.e., practicing the Mediterranean diet). 

In the present study, girls reported to have snacked more than boys have. This could be due to COVID-19 having brought stressful times [49], especially for younger girls [50]. In fact, the literature suggests that girls are more vulnerable to stress-induced eating [51]. Regarding the school grades, younger students from 5th-6th grades reported having snacked more than older students did, both from 7th-9th and 10th-12th grades. This is consistent with the literature reporting that the percentage of snack consumers decreases from six to 18 years old [14]. Moreover, we did not find differences in snack consumption frequency considering students’ income. This result is at odds with the literature, which has reported a positive relationship between low-income and snacking behaviors [18]. The current result could be due to the Portuguese governmental initiatives aimed to promote snack intake among students with lower SES (e.g., the Municipality of Arganil, provided free snacks to students from low SES backgrounds) [52]. Finally, regarding where the snack was acquired, in line with the literature, most students brought snacks from home at every observed moment [19].

Regarding the second goal, participants increased their consumption of whole fruit and total fruits during the second lockdown period; the mean of portions consumed dropped again after students returned to school. Again, this result is inconsistent with data from other cultures reported by the literature (e.g., Ruiz-Roso et al., Sidor and Rzymski [28,29]), but is aligned with the study by Rodríguez-Pérez et al. [31] conducted in Spain during the COVID-19 pandemic. The participants in the latter study reported adhering to a Mediterranean diet, including higher fruit consumption. This match may be due to Portugal and Spain sharing cultural aspects, among which a cultural call to engage in a Mediterranean diet. Regular fruit consumption at early ages was associated with higher academic scores [53], and is promoted by WHO [54] as a way to support both mental and physical health [55]. Thus, educators and practitioners could consider making further efforts to find ways to help students eat the recommended portions of fruit, particularly in crises such as lockdown periods. 

Still on the good side of the chain, during the second lockdown period, students decreased the consumption of refined grains, with the gains remaining over the post-second lockdown moment. These results are encouraging since refined grains consumption should be moderated, particularly when associated with cereal-based foods with high levels of added sugar and saturated fats, like cakes and biscuits [56]. Students of the current study also consumed lower added sugars during the second lockdown, compared to pre- and post-second lockdown moments. These results are inconsistent with some of the literature [29] but are in accordance with the study conducted in Spain [31]. The decrease in added sugars consumption during the lockdown period is an encouraging result since the WHO recommends low added sugars consumption for physical well-being [54], and the literature shows that sugar intake is associated with poor academic achievement [57]. Moreover, in the present sample, students also decreased their consumption of saturated fats during the second lockdown period, but increased their consumption in the post-second lockdown period. Furthermore, cultural norms seem to play a role in the consumption decrease in these components during the lockdown. In some countries, fast-foods rich in saturated fats were preferred during the lockdown [29]; however, in countries with similar eating patterns to the Portuguese ones, there was a reduction in pastries consumption [31]. Nevertheless, the reduction in saturated fat consumption during lockdown is a positive outcome since the consumption of saturated fat should be reduced to as low as possible due to its association with heart diseases, obesity, and cancer [58]. Lastly, present data also show that the consumption of fatty acids during the pre- and post-second lockdown was significantly higher than during the second lockdown moment. 

The factor that seemed to have weakened the quality of snacks during the second lockdown was the reduction in total protein foods consumption. Proteins have been proven to enhance memory, an important cognitive tool to improve academic performance [37]. Additionally, it is worth mentioning proteins’ qualities in preventing obesity, preserving lean body mass, and improving life quality [59]. However, this result is consistent with the literature reporting that adherence to the Mediterranean diet during lockdown [31] was associated with a reduction in protein intake. 

The current study presents promising results that may provide implications for practice and future research suggestions. First, breakfast consumption was highly prevalent among our sample in the distinct moments-consistently above 90%-including during the second lockdown. This result is very encouraging and suggests that future studies and interventions may want to focus exclusively on this subsample of students that do not take breakfast regularly. It would be interesting to learn whether they share some characteristics and understand why they do not have breakfast regularly. This could help inform future tailored interventions exclusively targeting this group. Second, in general, the content of the student’s snacks during the second lockdown seems to have increased in quality, being healthier than during regular school times (i.e., increased consumption of whole and total fruits, decreased consumption of refined grains, added sugars, saturated fats, and fatty acids). The literature shows that students are more likely to choose unhealthy snacks when influenced by peers [17], and during the lockdown, this influence diminished. Moreover, when students were at home, it is possible that the majority had time to prepare their snacks, the availability of unhealthy ones was lower [31,36], and did not need, or did not have the opportunity, to buy highly processed foods in school cafeterias and food outlets near the school. 

Healthy behavior promotion stakeholders should consider designing actions that enable students to maintain the improvements regarding snack content achieved during the lockdown throughout the school year. Following European best practices, policymakers could consider improving the school food environment by providing healthy foods in the school setting, for example, limiting unhealthy products in the school cafeterias and vending machines [60]. In addition, the school food environment could also be promoted outside the school setting; for example, by restricting advertisements of unhealthy food products and drinks (e.g., drinks with added sugars) [60]. Moreover, in the case of students that bring snacks from home, it is important to equip students with tools, such as self-regulation competencies, to help them prepare healthy lunch boxes (e.g., Pereira et al. [61]). For example, Tilley et al. [62] presented the healthy lunch box challenge. This challenge comprised goal setting and messages targeting positive recommended behaviors and rewards. This challenge is also a low-cost and innovative initiative likely to influence the content of children’s snacks positively. At policymaking level, the definition of a Nutri-Score (i.e., a logo that shows the nutritional quality of packaged food products, classifying them with five letters, from A to E, associated with colors that vary between dark green and red; dark green (A) is the healthiest option and red (E) is the unhealthiest option) as a mandatory Front-of-Pack Nutrition Label could also be an important strategy to help students make healthy snack choices. Some brands already use a Nutri-Score; however, Portugal has not yet adopted this Front-of-Pack Nutrition Label as mandatory for all products. The adoption of this measure would be useful as research suggest that Nutri-Score seems to be the most useful Front-of-Pack Nutrition Label compared to label components, such as reference intakes and multiple traffic lights. Adopting this measure would contribute to inform Portuguese consumers about the nutritional quality of foods and to help them in choosing healthy option for their snacks [63].

This study counts with some limitations that need to be acknowledged. First, the content of the snacks was reported by the school teachers, which could have led to incorrect or invalid responses. Future studies could consider using triangulation methods, such as photos of the content of students’ lunch boxes, for a more precise analysis. In fact, triangulation of methods (e.g., self-report questionnaires and photos) and respondents (e.g., teachers and students) may help to ensure that the data reflects with more trustworthiness what is being investigated (e.g., the real content of student’s snacks) [64]. Second, due to the dichotomic nature of some questions, it was not possible to access other relevant information. Particularly, the exact moment of the snack intake (i.e., morning break, afternoon break), how many snacks students had, and how they distributed the food among the distinct snacking moments. In the future, it could be interesting to analyze the consumption patterns across the day. Finally, on each data collection moment, students were only enquired once; this aspect limits the possibility of extrapolating data to the rest of the week or month. Future studies could consider using diaries to understand breakfast and snack consumption patterns throughout an entire period. 

## 5. Conclusions

Overall, the results of the present study contribute to the existing literature by shedding light on the changes in student’s breakfast and snack patterns in pre-, during, and post-second lockdown periods. Present results suggest that staying at home does not necessarily mean a decrease in the quality of students’ breakfast and snack patterns. In fact, the current study results indicated that most of the students: (i) consumed breakfast throughout the school year and maintained this habit during the second COVID-19 lockdown; (ii) decreased snack consumption during the second COVID-19 lockdown; and (iii) increased fruit consumption while decreasing the intake of pastries and sweets (e.g., refined grains, added sugars) in their snacks during this period. These data are similar to the ones from Spain, reinforcing the influence that culture plays in the development of eating patterns. These findings are expected to help inform the design of healthy behavior promotion actions, such as improving the school food environment and teaching children to prepare healthy lunch boxes. 

## Figures and Tables

**Table 1:** Codebook with the HEI components present in the data and correspondent examples.

HEI Components	Description	Examples from Data
Total Fruits	Includes all forms of fruit.	Fruit juice, marmalade
Whole Fruits	Includes intact fruit.	Banana, apple
Whole Grains	Includes grains containing the entire grain kernel, i.e., the bran, germ, and endosperm. The kernel can be intact, ground, or broken.	Whole grain bread
Dairy	Includes foods containing or made from milk.	Yogurt, cheese
Total Protein Foods	Includes high protein foods, including those of vegetal origin.	Ham
Seafood and Plant Proteins	Includes animals from the sea that can be eaten, especially fish or sea creatures with shells; includes legumes.	Soy, tuna
Fatty Acids	Includes animal and vegetal fats, mainly divided into saturated (i.e., solid fats) and unsaturated (i.e., oils).	Cereals, cereal bar
Refined Grains	Includes grains that do not contain all of the components of the entire grain kernel, i.e., the bran, germ, or endosperm.	White bread
Sodium	Includes any food high in salt.	Crackers
Added Sugars	Includes sugars that are added during the processing of foods (e.g., sucrose or dextrose), foods packaged as sweeteners (e.g., table sugar), sugars from syrups and honey, and sugars from concentrated fruit or vegetable juices.	Cookies
Saturated Fats	Includes fat that contains a high proportion of fatty acids-also known as solid fat because it is solid at room temperature.	Cake, butter

**Table 2:** Frequencies of the first goal—breakfast, snacking, and where the snack was acquired from analyses.

	T1 (Pre-)		T2 (Pre-)		T3 (during)		T4 (Post-)		T5 (Post-)	
Variables	Boys n (%)	Girls n (%)	Boys n (%)	Girls n (%)	Boys n (%)	Girls n (%)	Boys n (%)	Girls n (%)	Boys n (%)	Girls n (%)
Breakfast										
5th–6th Grade										
No	-	-	5(5.0%)	3(3.0%)	5(3.9%)	8(6.3%)	7(5.7%)	3(2.4%)	7(5.0%)	5(3.6%)
Yes	-	-	38(38.0%)	54(94.0%)	59(46.1%)	56(43.8%)	60(48.8%)	53(43.1%)	63(45.3%)	64(46.0%)
7th–9th Grade										
No	-	-	12(6.2%)	9(4.6%)	16(4.8%)	14(4.2%)	18(5.5%)	21(6.4%)	16(5.2%)	18(5.8%)
Yes	-	-	92(47.2%)	82(42.1%)	162(48.9%)	139(42.0%)	156(47.7%)	132(40.4%)	150(48.5%)	125(40.5%)
10th–12th Grade										
No	-	-	0(0.0%)	2(5.4%)	0(0.0%)	1(1.9%)	1(1.8%)	2(3.5%)	17(54.8%)	14(54.2%)
Yes	-	-	22(59.5%)	13(35.1%)	30(57.7%)	21(40.4%)	32(56.1%)	22(38.6%)	0(0.0%)	0(0.0%)
Total										
No	-	-	17(5.1%)	14(4.2%)	21(4.1%)	23(4.5%)	26(5.1%)	26(5.1%)	23(4.8%)	23(4.8%)
Yes	-	-	152(45.8%)	149(44.9%)	251(49.1%)	216(42.3%)	248(48.9%)	207(40.8%)	230(48.0%)	203(42.4%)
Snacking										
5th–6th Grade										
No	3(5.4%)	0(0.0%)	2(2.0%)	0(0.0%)	11(8.6%)	7(5.5%)	2(1.2%)	0(0.0%)	4(2.5%)	5(3.1%)
Yes	24(42.9%)	29(51.8%)	41(41.0%)	57(57.0%)	53(41.4%)	57(44.5%)	76(46.6%)	85(52.1%)	73(45.1%)	80(49.4%)
7th–9th Grade										
No	15(5.5%)	3(1.1%)	20(7.2%)	11(4.0%)	46(13.9%)	27(8.2%)	33(10.1%)	18(5.5%)	42(12.8%)	19(5.8%)
Yes	136(49.6%)	120(43.8%)	128(46.0%)	119(42.8%)	132(39.9%)	126(38.1%)	141(43.1%)	135(41.3%)	135(41.0%)	133(40.4%)
10th–12th Grade										
No	6(10.5%)	1(1.8%)	5(13.5%)	1(2.7%)	4(7.7%)	4(7.7%)	7(12.1%)	4(6.9%)	3(9.4%)	1(3.1%)
Yes	26(45.6%)	24(42.1%)	17(45.9%)	14(37.8%)	26(50.0%)	18(34.6%)	26(44.8%)	21(36.2%)	14(43.8%)	14(43.8%)
Total										
No	24(6.2%)	4(1.0%)	27(6.5%)	12(2.9%)	61(11.9%)	38(7.4%)	42(7.7%)	22(4.0%)	49(9.4%)	25(4.8%)
Yes	186(48.1%)	173(44.7%)	186(44.8%)	190(45.8%)	211(41.3%)	201(39.3%)	242(44.3%)	241(44.0%)	222(42.4%)	227(43.4%)
Brought from Home										
5th–6th Grade	20(37.7%)	27(50.9%)	43(42.6%)	53(52.5%)	-	-	52(37.4%)	65(46.8%)	56(37.1%)	67(44.4%)
7th–9th Grade	120(46.9%)	110(43.0%)	109(44.1%)	113(45.7%)	-	-	114(45.6%)	120(48.0%)	105(41.8%)	112(44.6%)
10th–12th Grade	23(46.0%)	23(46.0%)	14(45.2%)	14(45.2%)	-	-	20(46.5%)	20(46.5%)	12(44.4%)	13(48.1%)
Total	163(45.4%)	160(44.6%)	166(43.8%)	180(47.5%)	-	-	186(43.1%)	205(47.5%)	173(40.3%)	192(44.8%)
Bought on the School										
5th–6th Grade	1(1.9%)	2(3.8%)	0(0.0%)	1(1.0%)	-	-	3(2.2%)	5(3.6%)	5(3.3%)	6(4.0%)
7th–9th Grade	10(3.9%)	6(2.3%)	14(5.7%)	6(2.4%)	-	-	9(3.6%)	6(2.4%)	16(6.4%)	15(6.0%)
10th–12th Grade	2(4.0%)	1(2.0%)	3(9.7%)	0(0.0%)	-	-	3(7.0%)	0(0.0%)	1(3.7%)	0(0.0%)
Total	13(3.6%)	9(2.5%)	17(4.5%)	7(1.8%)	-	-	15(3.5%)	11(2.5%)	22(5.1%)	21(4.9%)
Brought and Bought										
5th–6th Grade	3(5.7%)	0(0.0%)	0(0.0%)	4(4.0%)	-	-	9(6.5%)	5(3.6%)	11(7.3%)	6(4.0%)
7th–9th Grade	6(2.3%)	4(1.6%)	5(2.0%)	0(0.0%)	-	-	1(0.4%)	0(0.0%)	2(0.8%)	1(0.4%)
10th–12th Grade	1(2.0%)	0(0.0%)	0(0.0%)	0(0.0%)	-	-	0(0.0%)	0(0.0%)	1(3.7%)	0(0.0%)
Total	10(2.8%)	4(1.1%)	5(1.3%)	4(1.1%)	-	-	10(2.3%)	5(1.2%)	14(3.3%)	7(1.6%)

**Table 3:** Post hoc comparisons of snacking frequency across the five moments (T1–T5).

	Moments (T)									
	T1 vs. T2	T1 vs. T3	T1 vs. T4	T1 vs. T5	T2 vs. T3	T2 vs. T4	T2 vs. T5	T3 vs. T4	T3 vs. T5	T4 vs. T5
Snacking Frequency	*ns*	<0.001	*ns*	0.003	<0.001	*ns*	*ns*	0.002	*ns*	*ns*

**Table 4:** Post hoc comparisons of snacking frequency according to the school cycle.

	School Cycle		
	5th–6th grade vs. 7th–9th grade	5th–6th grade vs. 10th–12th grade	7th–9th grade vs. 10th–12th grade
Snacking Frequency	***	*	*ns*

* *p* < 0.05; *** *p* < 0.001.

**Table 5:** Portions of each component consumed among moments (T1–T5), and results of the Repeated Measures ANOVA.

	Moments (T)					
	T1 (n = 125) Mean (*SD*)	T2 (n = 125) Mean (*SD*)	T3 (n = 125) Mean (*SD*)	T4 (n = 125) Mean (*SD*)	T5 (n = 125) Mean (*SD*)	
Total Fruits	0.29(0.49)	0.27(0.48)	0.46(0.58)	0.27(0.48)	0.18(0.41)	F (3.77, 467.93)
						7.26 ***
Whole Fruits	0.20(0.45)	0.14(0.34)	0.40(0.55)	0.14(0.34)	0.11(0.31)	F (2.81, 348.98)
						16.37 ***
Whole Grains	0.10(0.33)	0.10(0.30)	0.14(0.34)	0.10(0.35)	0.06(0.30)	F (3.64, 451.61)
						1.02
Dairy	1.02(0.76)	0.98(0.78)	0.98(0.81)	0.98(0.73)	0.96(0.82)	F (3.69, 457.32)
						0.17
Total Protein Foods	0.54(0.65)	0.45(0.56)	0.30(0.50)	0.38(0.56)	0.46(0.63)	F (3.71, 460.26)
						3.45 *
Seafood and Plant Protein	0.08(0.30)	0.08(0.27)	0.03(1.18)	0.05(0.21)	0.04(0.23)	F (3.46, 429.59)
						1.27
Fatty Acids	2.55(1.08)	2.42(1.07)	2.06(1.10)	2.38(1.03)	2.20(1.14)	F (3.77, 466.97)
						5.62 ***
Refined Grains	1.34(0.62)	1.21(0.51)	0.97(0.54)	1.22(0.57)	1.12(0.53)	F (3.78, 469.26)
						10.24 ***
Sodium	1.64(1.03)	1.58(1.13)	1.38(1.22)	1.59(1.01)	1.50(1.22)	F (3.8, 471.23)
						1.55
Added Sugars	2.38(1.05)	2.23(0.98)	1.66(0.99)	2.06(0.98)	2.14(0.99)	F (4, 496)
						11.97 ***
Saturated Fats	1.59(0.80)	1.48(0.81)	1.25(0.76)	1.52(0.74)	1.43(0.88)	F (3.89, 481.59)
						4.15 *

* *p* < 0.05; *** *p* < 0.001.

**Table 6:** Post hoc comparisons of the number of portions of each component consumed among moments (T).

	Moments (T)									
	T1 vs. T2	T1 vs. T3	T1 vs. T4	T1 vs. T5	T2 vs. T3	T2 vs. T4	T2 vs. T5	T3 vs. T4	T3 vs. T5	T4 vs. T5
Total Fruits	*ns*	*ns*	*ns*	*ns*	*	*ns*	*ns*	*	***	*ns*
Whole Fruits	*ns*	*	*ns*	*ns*	***	*ns*	*ns*	***	***	*ns*
Whole Grains	*ns*	*ns*	*ns*	*ns*	*ns*	*ns*	*ns*	*ns*	*ns*	*ns*
Dairy	*ns*	*ns*	*ns*	*ns*	*ns*	*ns*	*ns*	*ns*	*ns*	*ns*
Total Protein Foods	*ns*	*	*ns*	*ns*	*ns*	*ns*	*ns*	*ns*	*ns*	*ns*
Seafood and Plant Protein	*ns*	*ns*	*ns*	*ns*	*ns*	*ns*	*ns*	*ns*	*ns*	*ns*
Fatty Acids	*ns*	**	*ns*	*ns*	*	*ns*	*ns*	*ns*	*ns*	*ns*
Refined Grains	*ns*	***	*ns*	*	*	*ns*	*ns*	***	*ns*	*ns*
Sodium	*ns*	*ns*	*ns*	*ns*	*ns*	*ns*	*ns*	*ns*	*ns*	*ns*
Added Sugars	*ns*	***	*	*ns*	***	*ns*	*ns*	**	***	*ns*
Saturated Fats	*ns*	*	*ns*	*ns*	*ns*	*ns*	*ns*	*	*ns*	*ns*

* *p* < 0.05; ** *p* < 0.01; *** *p* < 0.001.

## Data Availability

Data are available from the corresponding author upon reasonable request.
